# Dense sampling reveals behavioral oscillations in rapid visual categorization

**DOI:** 10.1038/srep16290

**Published:** 2015-11-06

**Authors:** Jan Drewes, Weina Zhu, Andreas Wutz, David Melcher

**Affiliations:** 1 Center for Mind/Brain Sciences (CIMeC), University of Trento Corso Bettini 31, 38068 Rovereto TN, Italy; 2 School of Information Science, Yunnan University Cuihu Beilu, Kunming 650091, China; 3 Kunming Institute of Zoology Chinese Academy of Sciences, 32 Jiaochang Donglu, Kunming 650223, China

## Abstract

Perceptual systems must create discrete objects and events out of a continuous flow of sensory information. Previous studies have demonstrated oscillatory effects in the behavioral outcome of low-level visual tasks, suggesting a cyclic nature of visual processing as the solution. To investigate whether these effects extend to more complex tasks, a stream of “neutral” photographic images (not containing targets) was rapidly presented (20 ms/image). Embedded were one or two presentations of a randomly selected target image (vehicles and animals). Subjects reported the perceived target category. On dual-presentation trials, the ISI varied systematically from 0 to 600 ms. At randomized timing before first target presentation, the screen was flashed with the intent of creating a phase reset in the visual system. Sorting trials by temporal distance between flash and first target presentation revealed strong oscillations in behavioral performance, peaking at 5 Hz. On dual-target trials, longer ISIs led to reduced performance, implying a temporal integration window for object category discrimination. The “animal” trials exhibited a significant oscillatory component around 5 Hz. Our results indicate that oscillatory effects are not mere fringe effects relevant only with simple stimuli, but are resultant from the core mechanisms of visual processing and may well extend into real-life scenarios.

Our visual system is capable of rapidly extracting high-level meaning from a visual scene. Studies have shown that humans are able to discriminate visual category for scenes presented as briefly as 20 ms^1^,^2^ and to initiate actions contingent on target category in as little as 100 ms^2^,^3^. This ability to grasp the gist of a briefly presented visual scene can also occur when multiple images are presented in succession^4^, even at high rates of presentation^5^.

These findings raise critical question of how the visual system is able to quickly categorize a new, unexpected stimulus. It is commonly assumed that the ultra-fast mode of visual target identification is based on feed-forward processing^3^,^6^,^7^. However, other studies have shown evidence that recurrent processes also play an important role in object and scene processing and that categorization can involve temporal integration^8^,^9^,^10^,^11^. Moreover, it has been argued that recurrent processes may be required for stimuli to be consciously perceived^12^,^13^.

The goal of the present study was to investigate three critical issues regarding rapid visual categorization. The first was the temporal window during which information is combined in making an initial judgment of visual category. Previous studies have focused on the *minimum* duration necessary for categorization, but a full understanding of the underlying mechanisms would include also the temporal integration window (TW) during which additional information can affect perception. Second, we examined the role of recurrent processing in rapid visual categorization. One hallmark of recurrent processing is oscillations in behavioral performance, reflecting the interaction between the stimulus and the state of ongoing brain activity^14^. The perceptual system undergoes periods of optimal and sub-optimal performance, as shown in various perception tasks such as target detection^15^,^16^ and also in motor responses^17^,^18^,^19^,^20^. Finally, this study examined the claim that rapid categorization depends on target category, such that certain target categories, such as animals, are afforded a privileged kind of processing^21^,^22^,^23^,^24^,^25^,^26^.

To test these questions, we presented target images (animal or vehicle) in an RSVP sequence of natural scenes not containing any target objects (20 ms/50 Hz). We added two novel manipulations in order to investigate the mechanisms of rapid categorization. First, we inserted a bright flash into the rapid image sequence at a controlled, but randomized time (see methods below). This flash should induce a phase reset in the visual system, creating a degree of phase coherence across both trials and subjects^27^,^28^. By varying the onset of the target relative to the flash, it is possible to align and systematically sample the time course of performance in order to find signs of behavioral oscillations^16^,^29^,^30^,^31^,^32^.

In addition, we included trials in which there were two targets in the RSVP, separated by a varying ISI. We expected that presenting two targets immediately after each other, and within the temporal integration window, would lead to highest levels of performance and that lengthening the ISI would eventually eliminate most of the benefit of having two targets. We investigated the time course of the interaction between the two stimuli to look for evidence of recurrent processing. After a certain delay congruent with the duration of the feedback loop, a second presentation of the target should reinforce processing of the original stimulus and lead to an increase in target recognition/discrimination performance.

## Methods

### Participants

A total of 38 participants completed the experiment. All subjects reported to have normal or corrected-to-normal vision, and gave informed written consent. Experiments were conducted in accordance with the Declaration of Helsinki and approved by the University of Trento ethical committee.

### Stimuli

**Figure 1:**
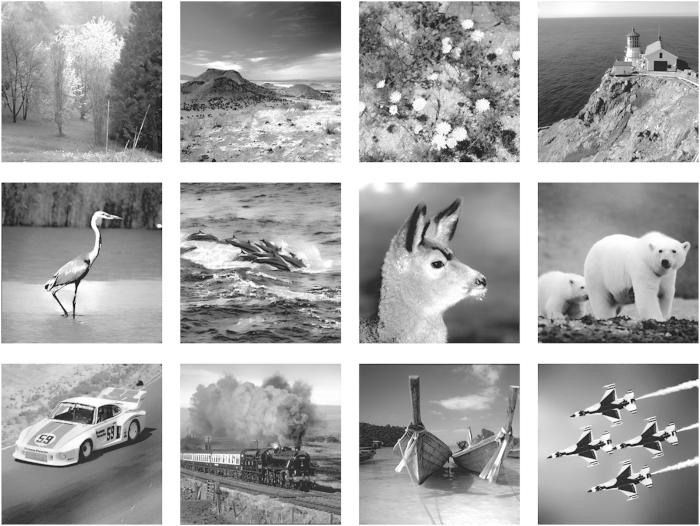
Sample images from the three categories (Copyright (c) 2015 Drewes, Zhu & Melcher and its licensors). All rights reserved. Top row: Neutral images (no animals or vehicles). Middle row: Animal images. Bottom row: Vehicle images.

Photographs of natural scenes were displayed in a rapid serial visual presentation (RSVP) paradigm. The images for use in this study were selected from the Corel Stock Photo Library and divided into 3 different categories. The first category was the “background/neutral” category. These images contained neither text, nor vehicles, nor animals, but otherwise any natural scene was deemed acceptable, resulting in a set of 3973 images. The other two categories were target images containing either an animal or vehicle (300 images each, see also^33^). As color information has been shown not to be critical for ultra-fast visual processing^33^,^34^,^35^, all images were converted to gray-scale. Sample images may be seen in Fig. 1. All images were square, measuring 300 pixels width and height, in 8 bit gray scales. To reduce low-level differences both within and across image categories, all images were histogram-equalized using the SHINE toolbox^36^.

### Experimental paradigm

**Figure 2:**
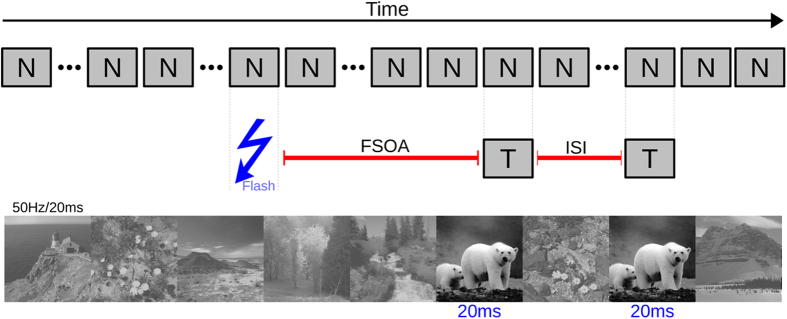
Illustration of the experimental paradigm. The underlying sequence of neutral images is shown as grey boxes labeled ‘N’. Timings of the points of insertion of the flash and the target stimuli are depicted within the RSVP stream of background (neutral) images. The timing of the first (or only) target with respect to the flash (FSOA) and the timing between the first and second target image (ISI) were randomized across trials (see Methods). Note that although this illustration shows two target images, some trials contained only a single target image (Images copyright (c) 2015 Drewes, Zhu & Melcher and its licensors).

A random stream of “background/neutral” images was displayed on a 20” CRT screen at 1024 × 768 pixels spatial resolution, with a vertical refresh rate of 100 Hz. Every image was shown for 2 refresh cycles, resulting in 50 Hz image frequency. Background/neutral images were displayed in the center of the screen, spanning approximately 10.8 deg of visual field, at 50% contrast surrounded by a black background. For the duration of one image (20 ms), at a randomized time ranging from 700 to 1200 ms after image sequence onset, the background of the screen was flashed from black to white. At a random time interval ranging from 200 to 740 ms (ten steps of 60 ms) thereafter (Flash/Stimulus onset interval), either one or two presentations of the same target chosen randomly from the animal or vehicle image categories were displayed (20 ms each), replacing the background/neutral image at the corresponding time point(s). When the target image was shown twice, the inter stimulus interval (ISI) between the two presentations was varied randomly in a systematic way between 0 and 600 ms (31 steps of 20 ms). Overall trial duration was fixed at 2.7 seconds. A flowchart of the paradigm can be seen in Fig. 2. After each trial, subjects were asked to indicate whether the target had been an animal or a vehicle by means of pressing one of two keys on the keyboard. Each block consisted of 16 repetitions of the single presentation condition and 4 repetitions of each of the 31 ISIs in the double presentation condition, with a total of 140 trials per block. Each experimental session consisted of 4 blocks, with a total of 560 trials. Prior to each block, the contrast of only the target images was adapted by a staircase procedure^37^ to level out subject performance at 57% correct based on those trials with a single target presentation. The experiment was programmed in Matlab, using the Psychtoolbox^38^.

## Results

On average, the QUEST-determined contrast for the target images was 71.1%, with a standard deviation across subjects of 7.2% and a standard error of 1.2%. Target discrimination performance in the single-presentation condition reached an average of 56.0%.

### Single target trials: Flash/Stimulus onset asynchrony (FSOA) analysis

**Figure 3:**
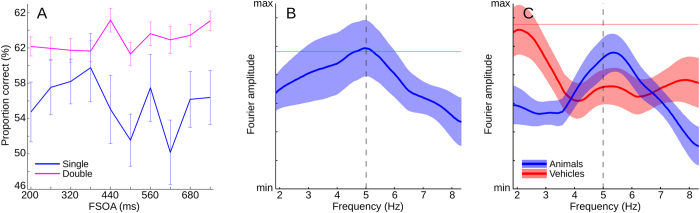
Flash/Stimulus onset asynchrony. (**A**) Performance correct in the target category discrimination task averaged across all ISIs, as a function of flash/stimulus onset asynchrony (N = 38, mean and 1 s.e.m.). Blue: single presentation trials only. Magenta: dual presentation trials only. (**B**) Fourier analysis of the behavioral performance from Panel (**A**). Single presentation trials only. Blue: mean and 1 s.e.m. of the amplitude spectra of the behavioral performance. Magenta line represents 5% margin of permutation test (N = 100k, Bonferroni corrected). Black, vertical dashed line marks location of the peak (5.03 Hz). (**C**) Fourier analysis, pooled across subjects, separate for target categories. Blue: amplitude spectra of behavioral performance with animal stimuli. Red: with vehicle stimuli (mean and 1 s.e.m). Vertical dashed line marks location of the peak from panel B).

Firstly, all trials were sorted by the temporal distance between the flash and the first (or only) stimulus presentation (Flash/Stimulus onset asynchrony, FSOA). Average target discrimination performance across all conditions ranged from 60.0 to 64.5%. When separating trials into single and double presentation, the average performance of the single presentation trials ranged from 50.2 to 60.0%, while the double presentation trials ranged from 62.0 to 65.0% (see Fig. 3A). The difference was significant (paired double-tailed t-test, p < 0.0001). To identify any periodic components relative to the flash onset in the time course of the single-presentation data, the individual means of each subject were centered, and the data were Fourier-transformed with the application of a hamming window and zero-padding. Of the resulting Fourier spectra, the amplitude information was averaged across subjects, while the phase information was discarded (for a similar approach, see^16^,^29^,^30^,^31^,^32^). The maximum of the resulting average spectrum was located at 5.03 Hz (see Fig. 3C). A zero distribution was then generated by randomly exchanging the individual time points of the subjects (permutation analysis, N = 100 k), with subsequent processing as before. Under the zero hypotheses that no periodic components exist, this should not change the average of the Fourier spectrum in a significant way. After sorting, the significance margin was determined by the percentage of zero distribution samples under the real averaged amplitude spectrum, as shown in Fig. 3C. The main peak in the spectrum was found to be significant (p < 0.05: 4.7–5.2 Hz, p_min_ = 0.0244 (5.03 Hz), Bonferroni corrected). Separating data by target category revealed no significant effect for either animal or vehicle stimuli (see Fig. 3D). When pooling both single and double presentation trials, a similar trend emerged, but did not reach significance. Also, no significant result was found in the double presentation trials alone, which we attribute to the additional variance from the variable ISI (see below).

### ISI Analysis

**Figure 4:**
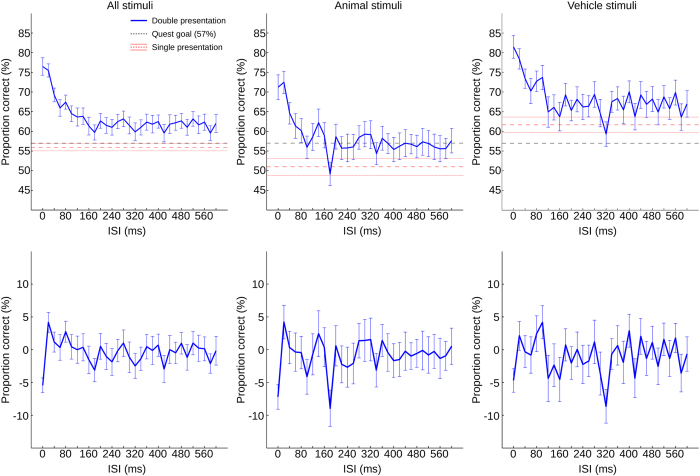
Double presentation analysis: behavioral data. Averaged across subjects (N = 38). Top row: raw data. Bottom row: data after centering and subtraction of decay function. Black dashed line: Quest goal of 57% performance. Red dashed line: average single-presentation performance (mean and 1 s.e.m.). Blue line: double presentation performance (mean and 1 s.e.m.). Left column: pooled over target categories. Middle column: animal targets only. Right column: vehicle targets only.

When sorting the double presentation trials by ISI, the average subject performance was best at short ISIs (maximum at 0ms ISI, 76.5% correct) and then decayed for longer ISIs (see Fig. 4, top row). Performance appears to decay and converge after around 120–160 ms, consistent with a temporal integration window of around 100 ms as has been found in other tasks (for review, see^39^). Nonetheless, the performance level remained significantly higher than in the single presentation condition, most likely due to probability summation (two independent chances to detect at least one target). Average performance with two targets beyond the temporal integration window (convergence level), as determined by the average over the range of 400–600 ms, was 64.8%.

The decay in performance for longer ISIs was found in both animal and vehicle trials. However, the overall level of discrimination performance differed between the two categories. The single presentation trials for the animal stimuli averaged 51.0% correct, while reaching 61.7% correct with the vehicle stimuli. For the dual presentation trials, the maximal performance for animal stimuli reached 72.5%, while reaching 81.5% with the vehicle stimuli. The apparent convergence level was determined by averaging the last 200 ms of the measurement interval, resulting in a convergence performance of 56.5% for the animal stimuli and 67.1% for the vehicle stimuli. To identify whether the convergence characteristics differed between stimulus classes, a decay function of the type 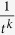 was chosen for a least-squares fitting approach, with *t* being the ISI and *k* being the exponent determining the decay. Under the assumption that the performance indeed converges to a fixed level at longer ISIs, all data was first centered by subtracting the average over the last 200 ms interval, separately for animal and vehicle trials. To achieve robustness against noise, a representative distribution was generated by repeatedly (N = 10000) sampling full sets (N = 38) of random subjects, and the decay function was fitted to the averages of the re-sampled sets. To minimize bias from compression, the resampled data was scaled to the common interval [0..1] prior to fitting. From this, distributions of exponentials *k* were collected for both animal and vehicle trials. A paired t-test then confirmed that the distributions were significantly different, with the vehicle distribution reaching convergence approximately 35% faster (mean and 95% confidence intervals: animals: 1.078 [0.766 1.542], vehicles: 1.242 [0.861 1.859], F(9999) = 47.77, p < 0.0001). When arbitrarily defining the convergence threshold (the point where convergence is considered to be achieved) at 10% (5%) distance from the convergence level, the time of convergence was determined to be on average 125 ms (309 ms) for animal stimuli and 81 ms (199 ms) for vehicle stimuli. This provides some tentative evidence that the integration window for animal stimuli might be longer than that for vehicle stimuli.

**Figure 5:**
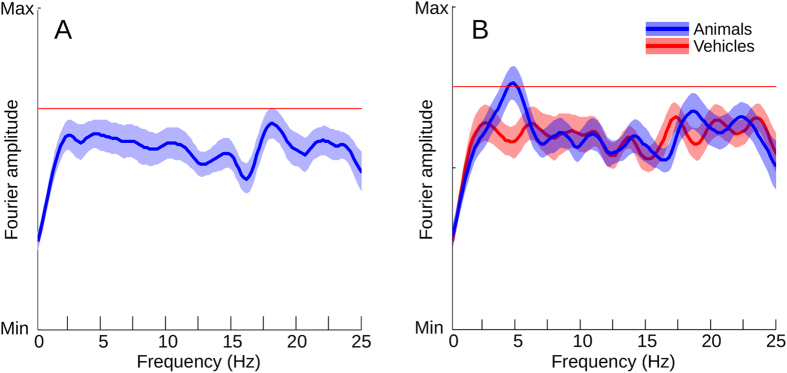
Double presentation analysis: Fourier spectrum. (**A**) Mean and 1 s.e.m across subjects and categories (N = 38). Red line illustrates significance threshold (Permutation test, N = 100K, n.s.). (B) Mean and 1 s.e.m. across subjects, separate for target categories. Blue: Animal targets. Red: Vehicle targets. Red line illustrates significance threshold (Permutation test, N = 100k, Bonferroni-corrected).

To identify possible oscillatory components in the ISI time course, any FSOA-related oscillation was removed from the ISI data by subtracting the average of the centered FSOA results. The average centered FSOA time course of the data was computed for each subject, and the result was subtracted from the individual trials depending on the respective FSOA timing. The data from each subject was then individually centered by subtracting the mean of the last 200 ms interval of the ISI time course. Afterwards, the data was fitted with a decay function (see above), which was then subtracted from the data to minimize spectral artifacts induced by the decay. The data was then considered centered (see Fig. 4, bottom row). Subsequently, the data were Fourier transformed, the amplitude spectra averaged, and the peak of the average determined. At the location of the peak, a permutation test was performed, similar to the one above (see Fig. 5). On the pooled data, the peak did not achieve significance. However, when separating trials by target category, a significant peak was identified with the animal trials at 4.88 Hz (p = 0.0074). No such peak was found with the vehicle stimuli; in fact a small trend in the opposite direction (trough, rather than peak) emerged at this frequency.

**Figure 6:**
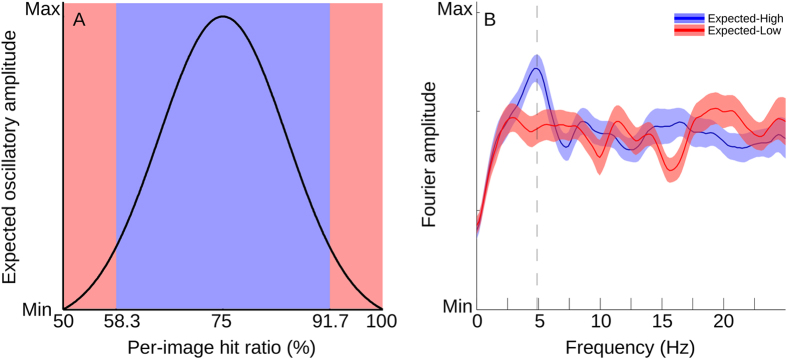
Distribution of oscillatory amplitude over stimuli. (**A**) Model assumption. Trials using images with near-chance (~50%) and near-perfect (~100%) hit ratio would be expected to contribute little to none to the evident oscillation. Trials using images with near-medium (~75%) hit ratio would be expected to contribute more to the oscillation. By extending a selection window symmetrically from 75%, images were divided into two equal subsets. The model predicts the blue subset (close to 75%) to result in stronger oscillatory amplitude than the red subset (close to 50%/100%). (B) Model verification. Computed oscillatory amplitude resulting from the expected-low and expected-high image groups. Blue: amplitude spectra of only those trials with expected-high images. Red: amplitude spectra of only those trials with expected-low amplitude. Mean and 1 s.e.m.

Assuming that the oscillation is stimulus-driven, the oscillatory amplitude associated with each stimulus image should be dependent on the average detection performance of that stimulus. If a stimulus is detected correctly on every trial (100% hit ratio), there will be no remaining variability to exhibit an oscillatory pattern – the oscillation would be squashed against the ceiling. In the opposite case, if an individual stimulus is detected only with chance performance, the oscillation would be squeezed against the floor. Consequently, oscillatory amplitude should therefore be strongest with stimuli that are detected with medium performance (about 75% hit ratio). To verify this, we first computed the average hit ratio across subjects for each animal stimulus. Each stimulus was then sorted into one of two equally sized groups (N = 150), depending on the difference of the average hit ratio achieved from the theoretical oscillatory optimum of 75% (see Fig. 6A). According to our hypothesis, the stimulus group with hit ratios close to 75% should result in larger Fourier amplitudes (Expected-High) than those with hit ratios close to 100% or 50% (Expected-Low). The mean of the resulting Fourier amplitudes of all 38 subjects, computed on each of the two stimulus groups separately, confirmed the hypothesis, as shown in Fig. 6B (Fourier amplitudes at 4.88 Hz: Expected-High, 1.22 ± 0.07; Expected-Low, 0.91 ± 0.08; mean ± 1s.e.m., Paired t-test: F(37) = 2.87, p = 0.0067).

In general, discrimination performance was higher with the vehicle stimuli than with the animal stimuli (72.5% vs. 81.5% at maximum, 56.5% vs. 67.1% at convergence). It may be that the vehicle stimuli were in fact easier to detect in the context of the neutral background image sequence; However, in a Continuous Flash Suppression (CFS) paradigm comparing these identical vehicle and animal images, no such difference was found in target detectability between classes^33^. The difference in performance may therefore reflect a decision bias for this specific task.

## Discussion

We employed a novel high-resolution RSVP-based dual-presentation paradigm to analyze the temporal dynamics of rapid visual object discrimination. Humans are known to be able to recognize the content of very briefly presented natural scenes^2^, even when images are presented in very rapid succession^4^,^5^. While these very rapidly presented scenes may not usually be consolidated in long-term memory^40^, our results do suggest that, at least for a brief period, the stimulus is represented in a brief (iconic/sensory) memory, as the detection/discrimination performance is significantly higher during the first 120–160 ms of ISI, allowing for temporal integration. At a later time, this boost in performance dissipates, possibly because the intense masking effect of the ongoing background RSVP causes the iconic/sensory memory to decay. At longer time intervals, the performance converged on a level that may represent simple probability summation: at larger ISIs the performance increase relative to the single presentation trials may be the result of two independent chances to perceive the presented target.

The main finding of the present study is that we were able to identify two oscillatory signatures in behavioral performance for natural scenes. First, for single-target trials, we found a 5 Hz oscillation that was time locked to the Flash-Stimulus onset asynchrony (FSOA). A regular oscillation in perceptual threshold (independent of target category) may explain this peak in the Fourier spectrum, as has been reported with other visual paradigms at varying frequencies from 4 to 11 Hz^14^,^15^,^16^,^29^. Our results show that the timing of this effect was aligned with the flash, which indicates that the flash displayed during our paradigm was capable of phase-resetting relevant rhythms in the brain.

Second, for double-target trials, we report a second 5 Hz oscillation in the dense sampling of performance for two animal targets as a function of the ISI between those two targets. In other words, participants were best at the task when the temporal separation of the first and second presentation of the target was in a multiple of around 200 ms, consistent with the idea of a “perceptual moment” in which information is combined^41^. The reason why we were successful in elucidating these oscillatory effects is to be found in the design of our paradigm. Firstly, we introduce a phase reset at a known time, which enables us to sort trials by their temporal distance from the phase reset. Secondly, we used a comparatively fine timescale in a dense sampling approach^16^,^29^,^30^,^31^,^32^. Lastly, the tuning of the task difficulty to almost (but not quite) breaking point adjusted most trials to an optimal point which made the presence of oscillations in performance manifest.

It is interesting to note that the first 5 Hz (5.03 Hz) oscillatory signature (linked to the flash) was found only in the pooled data, while the second 5 Hz (4.88 Hz) oscillatory activity (linked to the first presentation of the target) was found only with the animal stimuli (see Figs 3 and 5). There are at least two possible reasons for this difference. The first, less theoretically interesting, possibility is a decision bias. In a 2AFC task, subjects are asked to decide between two alternatives (“was it A or was it B?”). This question can however be solved with a proxy task, by making a binary decision on just one of the two alternatives (“was it A or not? For if it was not A, it must have been B.”). For example, our subjects could perform the task “Did I see an animal or not?” rather than “Did I see an animal or a vehicle?”. If most subjects also employed a conservative strategy, then this would create a decision bias towards the vehicle stimuli, consistent with the different average hit ratios of the two stimulus groups (Fig. 4). More importantly, our subjects would have been comparing the visual impressions to only one internal decision criterion (“animal”) rather than two.

A second explanation would be that animal images are indeed special to the human visual system, as has been previously suggested^21^,^22^,^23^,^24^,^25^,^26^. In this case, it would be possible for the feedback-driven oscillatory effect to be much stronger for the animal stimuli because a dedicated neural mechanism for vehicle stimuli simply may not exist, or a mechanism exists only in a more general, more variable or otherwise different fashion that was not revealed in our analysis.

Recurrent processing would be one plausible neural mechanism to explain this pattern of results. The first presentation of the animal target is processed along the ventral pathway. At some point along this processing path, feedback is generated and sent back to the earlier visual processing stages. If this feedback information arrives (for example at V1/V2) at precisely the same time as the new visual information resulting from the second target presentation, this second wave of information may optimally combine with the feedback from the first wave, resulting in an increased chance of successful target recognition. If the relevant feedback was generated only if the first target was already regarded by the visual system as a potential animal target (selective feedback), then such a feedback-driven oscillatory signature would only manifest itself with animal stimuli, not with vehicle stimuli. This temporally selective increase in task performance may then result in an oscillation of behavioral performance, as revealed in the time course of the behavioral performance recorded from our subjects. Such recurrent processing would be consistent with previous findings^11^,^12^, including scene processing^8^,^10^,^42^,^43^. This interpretation is also consistent with the idea of “perceptual echoes”, in which the presentation of a stimulus shows effects at regular intervals in later time periods in an oscillatory fashion^44^.

Indeed, the proposal that perceptual cycles play a critical role in temporal integration of multiple samples of a stimulus, due to recurrent processing, provides a theoretical motivation for behavioral and neural oscillations in the theta range in humans. Each new sample of the world involves feedforward and re-entrant processing. In natural viewing, we sample the world via eye movements^45^, hand movements^46^ or shifts in attention^29^,^30^,^31^,^32^ with an overt sampling of the world at a rate of 3–5 times per second^3^,^39^,^45^. Using a flash to reset and align these oscillations in the laboratory setting (as in the flash condition here) is a useful methodology to uncover these fluctuations, but in natural viewing oscillations would more likely be tied to perceptual sampling, via feedforward and re-entrant processing. In the case of the dual stimulus paradigm used here, performance on a given trial would vary depending on whether the first stimulus evoked a change in oscillatory activity (related to a phase reset) or whether the two stimuli fell into the same perceptual cycle (without a phase reset).

In terms of alternative explanations for this data, we can exclude the role of an “attentional blink” or priming. Behavioral performance when two targets must be independently reported has been shown to exhibit an attentional blink approximately 180–450 ms after a first stimulus presentation^47^,^48^, during which detection/identification of a second target is severely impaired. This effect appears to be most pronounced when the presented targets are spatially and featurally similar^49^. In our paradigm however, we would not expect to find an attentional blink, since the second target was identical to the first and discrimination between the two target presentations was never required. To the contrary, most likely the temporal integration of the first and second target presentation was responsible for the initial increase in discrimination performance during the first 120–160 ms.

The overall pattern of results also speaks against a more general priming effect as the underlying mechanism. While oscillatory activity in a compatible frequency range has been shown in priming paradigms^30^, our results differ in that we also obtained an oscillatory signature with the single-presentation condition, in which the flash could only serve as a neutral prime. However, Huang *et al.* found no oscillatory activity with neutral primes. Additionally, there would be no obvious reason why such priming-induced oscillations should only appear with animal stimuli, but not vehicles.

In summary, these results suggest oscillatory dynamics in the detection of real-world stimuli. Perceptual systems must solve the problem of how to create discrete objects and events out of a continuous flow of sensory information (for review, see^39^). Our findings are consistent with the idea that perceptual systems solve this problem by discretizing sensory input into perceptual units or cycles, alternating between states of higher and lower sensitivity to new input, and allowing for recurrent processing. The current findings indicate that such oscillatory effects are not mere fringe effects relevant only with simple stimuli, but instead are resultant from the core mechanisms of visual processing and may well be manifest even in real-life scenarios with natural scenes.

## Additional Information

**How to cite this article**: Drewes, J. *et al.* Dense sampling reveals behavioral oscillations in rapid visual categorization. *Sci. Rep.* **5**, 16290; doi: 10.1038/srep16290 (2015).
